# Characterization of distinct molecular interactions responsible for IRF3 and IRF7 phosphorylation and subsequent dimerization

**DOI:** 10.1093/nar/gkaa873

**Published:** 2020-10-22

**Authors:** Louise Dalskov, Ryo Narita, Line L Andersen, Nanna Jensen, Sonia Assil, Kennith H Kristensen, Jacob G Mikkelsen, Takashi Fujita, Trine H Mogensen, Søren R Paludan, Rune Hartmann

**Affiliations:** Department of Molecular Biology and Genetics, Aarhus University, 8000 Aarhus, Denmark; Department of Molecular Biology and Genetics, Aarhus University, 8000 Aarhus, Denmark; Department of Biomedicine, Aarhus University, 8000 Aarhus, Denmark; Department of Molecular Biology and Genetics, Aarhus University, 8000 Aarhus, Denmark; Department of Molecular Biology and Genetics, Aarhus University, 8000 Aarhus, Denmark; Department of Biomedicine, Aarhus University, 8000 Aarhus, Denmark; Department of Biomedicine, Aarhus University, 8000 Aarhus, Denmark; Department of Biomedicine, Aarhus University, 8000 Aarhus, Denmark; Institute for Frontier Life and Medical Sciences, Kyoto University, Kyoto 606–8507, Japan; Department of Biomedicine, Aarhus University, 8000 Aarhus, Denmark; Department of Infectious Diseases, Aarhus University Hospital Skejby, 8200 Aarhus, Denmark; Department of Clinical Medicine, Aarhus University, 8000 Aarhus, Denmark; Department of Biomedicine, Aarhus University, 8000 Aarhus, Denmark; Department of Molecular Biology and Genetics, Aarhus University, 8000 Aarhus, Denmark

## Abstract

IRF3 and IRF7 are critical transcription factors in the innate immune response. Their activation is controlled by phosphorylation events, leading to the formation of homodimers that are transcriptionally active. Phosphorylation occurs when IRF3 is recruited to adaptor proteins via a positively charged surface within the regulatory domain of IRF3. This positively charged surface also plays a crucial role in forming the active homodimer by interacting with the phosphorylated sites stabilizing the homodimer. Here, we describe a distinct molecular interaction that is responsible for adaptor docking and hence phosphorylation as well as a separate interaction responsible for the formation of active homodimer. We then demonstrate that IRF7 can be activated by both MAVS and STING in a manner highly similar to that of IRF3 but with one key difference. Regulation of IRF7 appears more tightly controlled; while a single phosphorylation event is sufficient to activate IRF3, at least two phosphorylation events are required for IRF7 activation.

## INTRODUCTION

The initial defense against a viral infection depends on the ability of the innate immune system to detect the invading pathogen via pattern recognition receptors (PRRs). Detection of viral pathogens is mediated through three main pathways: the endosomal Toll-like receptors (TLRs)—TRIF/MyD88, the cytosolic cyclic GMP-AMP synthetase (cGAS)—STING and the cytosolic RIG-I like receptors (RLR)—MAVS pathway (1–4). Upon recognition, the unique downstream adaptor proteins TRIF, STING and MAVS all mediate activation of the transcription factors interferon (IFN) regulatory factor 3 (IRF3) and IRF7.

The family of transcription factors known as interferon regulatory factors (IRFs) was first identified by their ability to induce the IFNβ promoter (5,6). The family contains nine members (IRF1–9) and the different members play key roles throughout the immune system. IRF3 and IRF7 are members of this family and are central in the production of type I (IFNα/β) and type III IFN (IFNλ) (7). Both IRF3 and IRF7 consist of a DNA-binding domain (DBD) that recognizes elements in IRF3/7 responsive promoters and a C-terminal regulatory domain (RD).

The RD of IRF3 plays a key role in the activation of IRF3 and facilitates the recruitment of the important cofactor CBP/P300 (8). Crystallographic studies of IRF3 RD demonstrated that this domain can be subdivided into two structurally distinct domains. The larger N-terminal domain (also referred to as the IRF association domain or IAD) consists of a β-sandwich of 5 and 6 antiparallel β-sheets. The β-sandwich is flanked by a bundle of helices on one end and a group of loops connecting a long helix that forms a positively charged surface on the other end (9,10). The C-terminal domain (also referred to as autoinhibitory domain or AD) is located as an extension of the bundle of helices in the N-terminal domain and consists of an alpha helix connected to the N-terminal domain by a loop. Both the positively charged helix and the C-terminal domain play key roles in the activation of IRF3 and are situated at opposite ends of the β-sandwich. The C-terminal domain is phosphorylated in response to virus infection (11–13) and contains two distinct and potentially important phosphorylation sites. The 2S site contains residue S385 and residue S386 (14) and the 5ST site contains residues S396, S398, S402, T404 and S405 (15,16). Contradicting data exist as to which of these phosphorylation sites are critical for IRF3 activity. On one hand, phosphorylation of S386 was demonstrated to be essential for IRF3 activation since mutation of this residue abolished all IRF3 activation (13). On the other hand, the 5ST site may also play an important role since substitution of the 5ST residues with the phosphomimic aspartic acid resulted in a constitutively active phenotype (15).

A simple model for IRF3 activation exists. This model suggests that docking of IRF3 to an activated adaptor protein positions IRF3 for phosphorylation in the C-terminal domain (17) leading to the formation of a homodimer (8,12) and the dimeric form translocates to the nucleus where it activates transcription (15,18). The adaptor molecules each harbor a common motif of pLxIS (p = hydrophilic amino acid, x = any amino acid) and the key serine in this motif becomes phosphorylated upon viral infection (S442, S366 and S210 for MAVS, STING and TRIF, respectively) (17). The activated adaptors are able to recruit IRF3 by forming an electrostatic interaction between the phosphorylated pLxIS motif on the adaptor and the positively charged surface in the N-terminal domain of IRF3 RD. The current model suggests that IRF3 interacts in an identical manner with the three adaptor proteins. However, this simple model is challenged by some of our recent data. We identified a mutation (R285Q) in a patient suffering from herpes simplex encephalitis (HSE). This mutation is located within the positively charged surface of IRF3 described above. A mutation in IRF3 affecting its function would normally lead to a global defect in innate immunity toward virus infection. However, the patient had no prior history of repeated infections with RNA viruses and we could subsequently show that the R285Q mutation had a milder effect on IFN responses in cells stimulated with Sendai virus (SeV) or influenza A virus compared to herpes simplex virus 1 (HSV-1) (19). Thus, the observed effect of the IRF3 surface mutation R285Q challenges the concept of a common IRF3 interaction mechanism utilized by the three innate immune adaptors.

Upon docking to adaptor proteins and subsequent phosphorylation, IRF3 is required to form a dimer in order to be transcriptionally active. Formation of the dimer leads to IRF3 translocation to the nucleus and a recent study demonstrated that IRF3 dimerization also leads to activation of the histone acetylase activity of the IRF3 co-factor CBP/P300 (20). A dimeric structure of the RD domain of IRF3 using the phosphomimic glutamic acid at both position 386 and 396 (S386E, S396E) exists (21). This work gave structural insight into how the IRF3 dimer is formed after phosphorylation but did not differentiate between the roles of phosphorylation at S386 and S396. Based on structural information, the IRF3 dimer is formed by interaction between the phosphoserines of the C-terminal domain of one IRF3 monomer and the positively charged surface in the N-terminal domain of another IRF3 monomer (9,10). Thus, the positively charged surface on IRF3 has a dual function of both adaptor docking and dimerization, although the molecular mechanism governing the individual steps in IRF3 activation is still not fully understood.

Here we show that the surface residues of IRF3 have distinct roles in docking to the adaptors and formation of the dimer, with R285 being involved in the docking of IRF3 to the innate immune adaptors, especially to STING, whereas R211 plays a key role in formation of the IRF3 dimer through interaction with phosphorylated S386. In analogy to the scenario described for IRF3, we show that IRF7 can be activated via interaction with MAVS and STING. Our data suggest that IRF7 is activated in a manner highly similar to that seen for IRF3, although activation of IRF7 appears more stringent than activation of IRF3.

## MATERIALS AND METHODS

### Lentiviral transduction and stimulation of IRF3-deficient THP-1-derived monocytes

Lentiviral vector constructs pCCL/PGK-V5-IRF3 were used to generate pCCL/PGK-V5-IRF3 (R211Q), pCCL/PGK-V5-IRF3 (R285Q) and pCCL/PGK-V5-IRF3 (R361Q). Packaging plasmids pMD2.G, pRSV-Rev and pMIDg/pRRE were transfected together with either pCCL/PGK-GFP, pCCL/PGK-V5-IRF3, pCCL/PGK-V5-IRF3 (R211Q), pCCL/PGK-V5-IRF3 (R285Q) or pCCL/PGK-V5-IRF3 (R361Q) into HEK293T cells using calcium phosphate transfection. Supernatants containing lentiviral particles were harvested and filtered through a 0.45-μm filter before adding polybrene to a final concentration of 8 μg/ml. The supernatants were added to IRF3-deficient THP-1 cells in a concentration of 1 × 10^5^ cells/ml in RPMI 1640 and passaged for 5 days before usage. IRF3-deficient THP-1 cells were seeded at 5 × 10^5^ cells/ml in 12-well dishes and differentiated into macrophages by addition of 100 nM phorphol myristate acetate (PMA). After 24 h, the medium was changed, and the cells were left another 24 h before further stimulation. The cells were stimulated for 6 h with either SeV (strain Cantell, 2 HAU/well), HSV-1 (strain 17+, MOI 9) or dsDNA (1 μg/ml) transfected into the cells using Lipofectamine 2000 (Life Technologies) according to manufacturer’s instructions. After the completed incubation period the cells were lysed, and the lysates subjected to RNA purification.

### RNA purification

E.Z.N.A.® Total RNA Kit I (Omega) was used for RNA purification following the manufacturer’s instructions. cDNA was synthesized from 1 μg RNA using Random Hexamer primer (Thermofisher Scientific) and RevertAid Reverse Transcriptase (Thermofisher Scientific).

### RT-qPCR

mRNA expression levels of *IFNB1* and *GAPDH* were detected by real-time quantitative PCR using SYBR green I (Roche) on Roche Lightcycler 480 II. For IFNβ, the following primers were used: *IFNB* forward 5′-TGGGAGGATTCTGCATTACC-3′ and *IFNB* reverse 5′-AAGCAATTGTCCAGTCCCAG-3′. For GAPDH the following primers were used: *GAPDH* forward 5′-CGACCACTTTGTCAAGCTCA-3′ and *GAPDH* reverse 5′-GGTGGTCCAGGGGTCTTACT-3′. Expression levels of V5-tagged IRF3 was detected using the following primers: *V5-IRF3* forward 5′-TCCTCGGTCTCGATTCTACG-3′ and *IRF3* reverse 5′-GGCCTGGAAGATTCCGAAAT-3′. Samples were analyzed in triplicates and relative mRNA levels were calculated using the formula: 2Δ*C*t (control − sample). Control *C*t values were generated as a mean of GAPDH values.

### Cell lines

IRF3-deficient and MAVS-deficient HEK293T cells were a kind gift from Veit Hornung and were cultured in DMEM (Sigma-Aldrich) supplemented with 10% FBS (Sigma-Aldrich), 100 U/ml penicillin (Sigma-Aldrich), 100 mg/ml streptomycin (Sigma-Aldrich) and 5 μg/ml plasmocin (Invitrogen). IRF3-deficient THP-1 cells were also a gift from Veit Hornung and were cultured in RPMI 1640 supplemented with 10% FBS (Sigma-Aldrich), 200 mM L-glutamine, 100 U/ml penicillin (Sigma-Aldrich), and 100 mg/ml streptomycin (Sigma-Aldrich). All cell lines were and maintained at 37°C with 5% CO_2_. Knockout cells were generated as described in (19).

### Plasmids

The IRF3, IRF7, MAVS and STING mutant plasmids were generated by site-directed mutagenesis using PfuUltra II Fusion DNA polymerase (Agilent) following the manufacturer’s instructions. The mutants were generated with either a pcDNA3.1/V5-IRF3, pcDNA3.1/V5-IRF7, pcDNA3.1/STING or pEF-BOS/MAVS-Flag-6xHis vector as template for the HEK293T cell assay or a pCCL/PGK-V5-IRF3 vector as template for the lentiviral production.

### Luciferase assay

**Figure 1.**
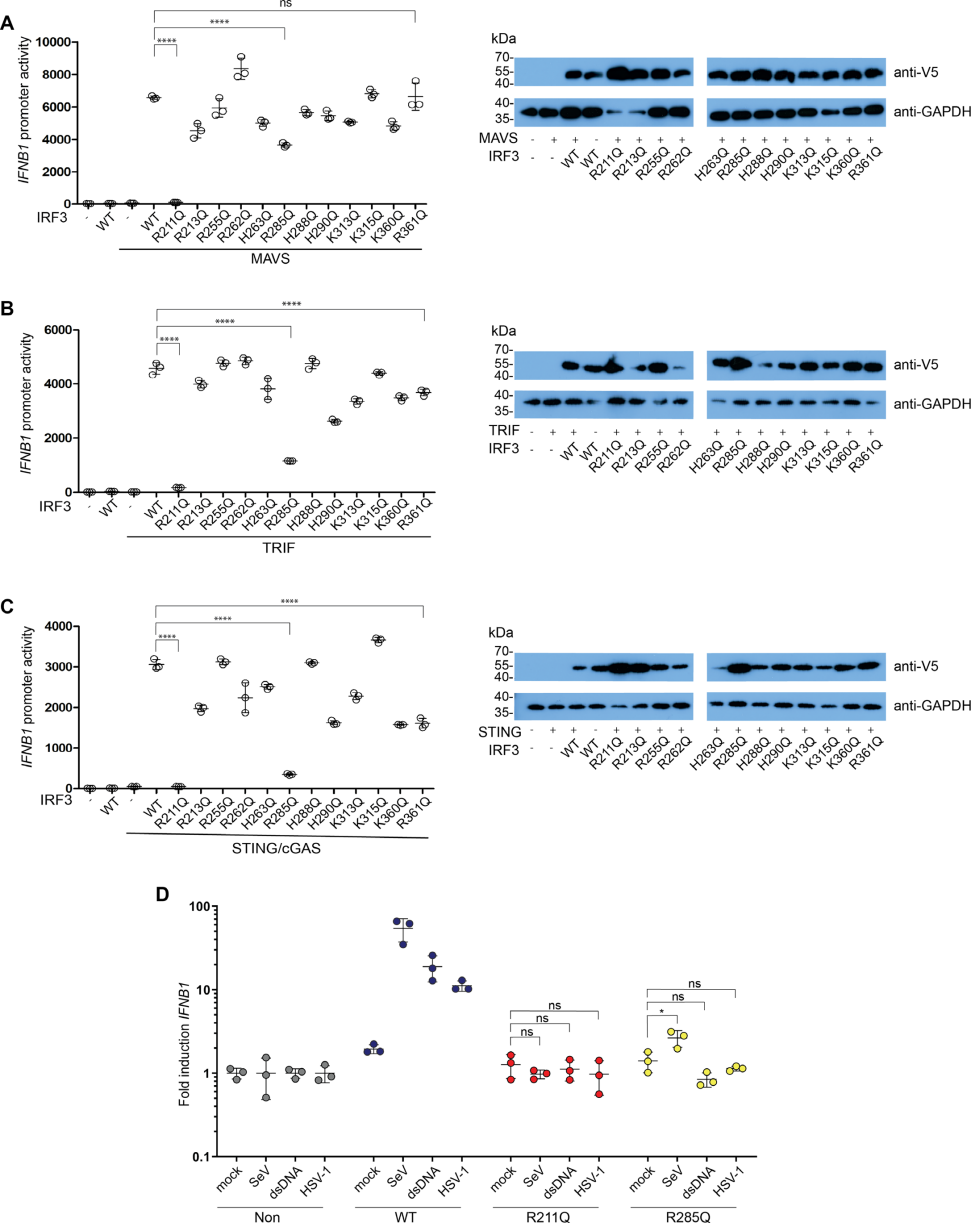
Identification and validation of IRF3 mutants with impaired activation. (**A–C**) IRF3-deficient HEK293T cells were transiently transfected with *IFNB1* promoter firefly luciferase reporter, a constitutively active *Renilla* luciferase reporter and either WT IRF3 or mutant IRF3 as indicated. The cells were stimulated with MAVS (A), TRIF (B) or STING and cGAS (C) and luciferase activities were measured 24 h post transfection. Firefly luciferase activity was normalized to *Renilla* luciferase activity and presented as triplicates ± SD. Similar data were obtained for three independent experiments (A+B) and for two independent experiments (C) (Supplementary Figure S1). (A–C) IRF3 and GAPDH amounts were detected by western blotting using an anti-V5 antibody for the detection of IRF3 and an anti-GAPDH antibody for the detection of GAPDH as a loading control. (**D**) IRF3-deficient THP-1 cells transduced with either IRF3 WT, IRF3 R211Q or IRF3 R285Q were infected for 6 h with SeV (2 HAU/ml) or HSV-1 (MOI 9) or stimulated for 6 h with 1 μg dsDNA. Total RNA was harvested and subjected to RT-qPCR for measurement of *IFNB1*. *IFNB1* mRNA levels were calculated relative to *GAPDH* expression and normalized to the non-transduced cells for each stimulation and is shown as triplicates ± SD. Similar data were obtained for two independent experiments. (A–D) One-way ANOVA test was used for statistical analysis. ns, not significant; *, P ≤ 0.05; ****, *P* ≤ 0.0001.

**Figure 2.**
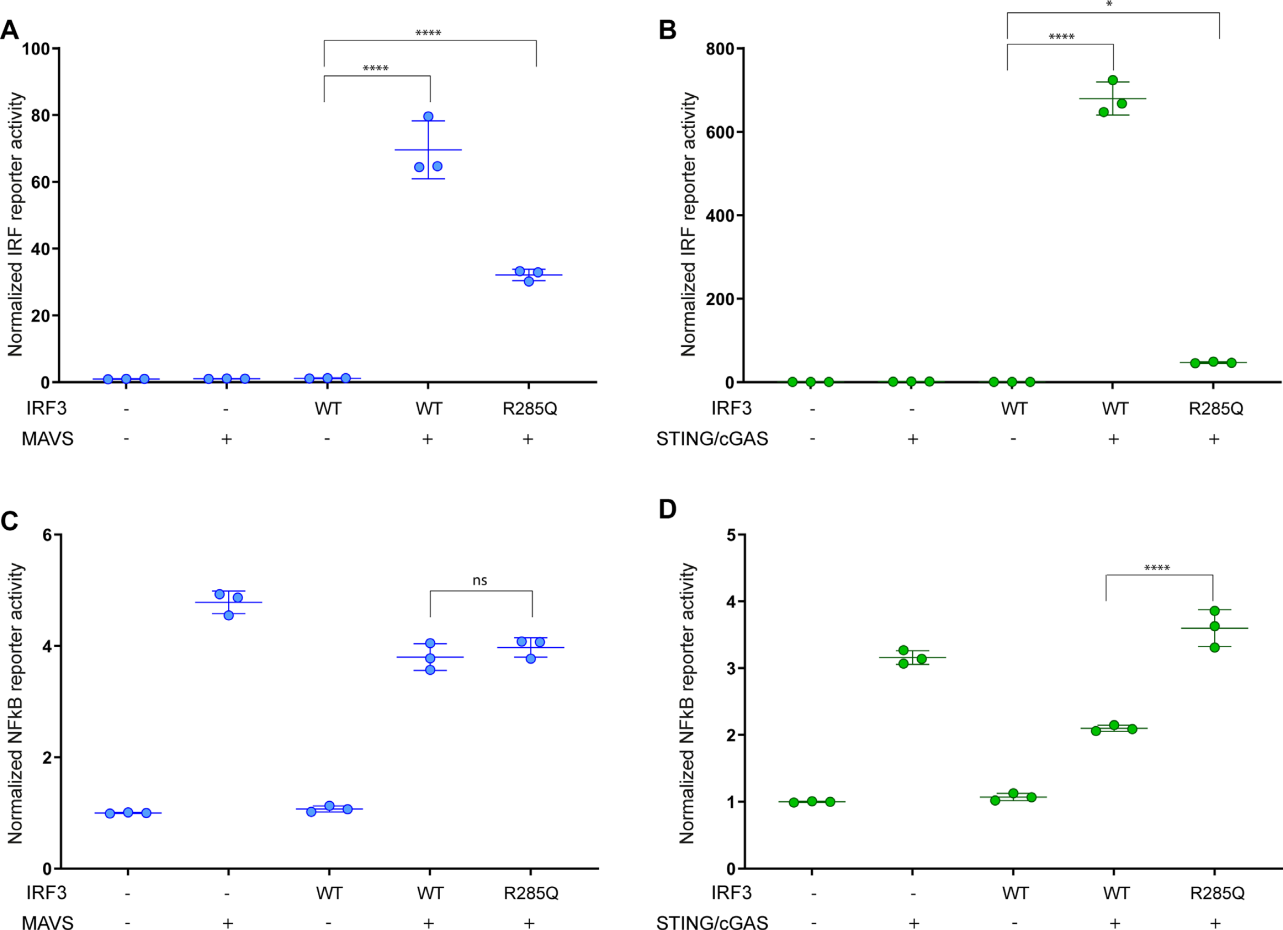
MAVS mediated NF-kB activation does not account for the differential effect of IRF3 R285Q. IRF3-deficient HEK293T cells were transfected with either a firefly luciferase reporter fused to 55 base pairs of the IFNB1 promoter followed by several repeats of IRF3 binding sites (**A** and **B**) or a firefly luciferase reporter fused to several repeats of NF-kB-binding sites (**C** and **D**). The cells were transfected with either IRF3 WT or R285Q and stimulated with MAVS (A and C) or STING and cGAS (B and D). Luciferase activities were measured 24 h post transfection and calculated relative to a constitutively active *Renilla* luciferase reporter. The data were normalized to the mock sample and presented as triplicates ± SD. Similar data were obtained for three independent experiments. One-way ANOVA test was used for statistical analysis; ns, not significant; *, *P* ≤ 0.05; ****, *P* ≤ 0.0001. Protein amounts were analyzed by western blotting (Supplementary Figure S3).

**Figure 3.**
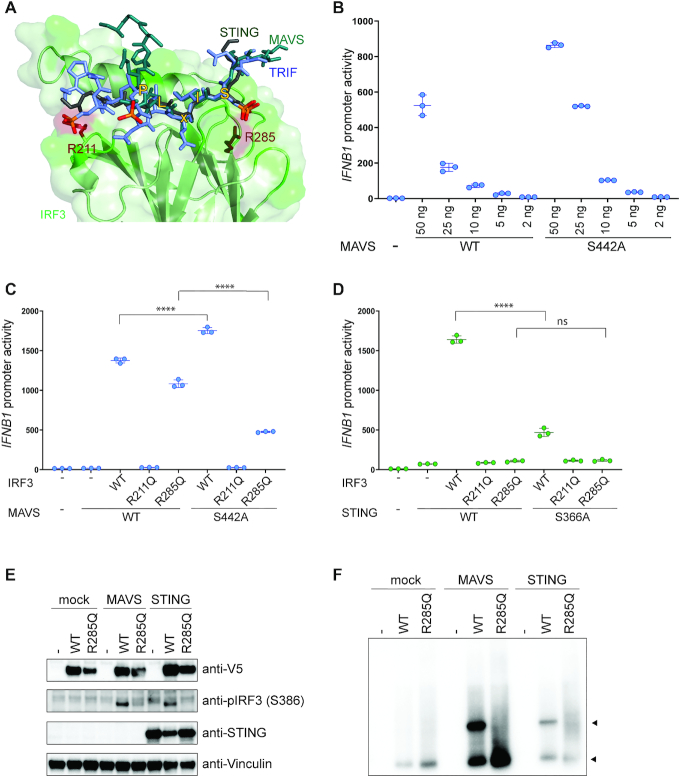
The role of residue R285Q in IRF3 docking to the adaptors MAVS and STING. (**A**) Structural representation of IRF3 bound to adaptor peptides. IRF3 is shown in green, MAVS in teal, TRIF in purple and STING in dark gray. Phosphorylation of the adaptor peptides is indicated in orange and red. IRF3 R211 and R285 are shown in dark red and the residues of the pLxIS motif are indicated with yellow. Figure was modeled on PDB ID: 5JEJ (pSTING/IRF3), 5JEK (pMAVS/IRF3, 5JEL (pTRIF/IRF3). (**B**) MAVS-deficient HEK293T cells were transiently transfected with *IFNB1* promoter firefly luciferase reporter, a constitutively active *Renilla* luciferase reporter and different amounts of MAVS WT or MAVS S442A as indicated and lysed after 24 h. (**C** and **D**) IRF3-deficient HEK293T cells were transiently transfected with *IFNB1* promoter firefly luciferase reporter, a constitutively active *Renilla* luciferase reporter and either WT IRF3 or mutant IRF3 as indicated. The cells were stimulated with either MAVS WT or MAVS S442A (B) or cGAS and either STING WT or STING S366A (C) and luciferase activities were measured 24 h post transfection. (B–D) Firefly luciferase activity was normalized to *Renilla* luciferase activity and presented as triplicates ± SD. One-way ANOVA test was used for statistical analysis. ns, not significant; ****, *P* ≤ 0.0001. Similar data were obtained for three independent experiments and protein expression was measured by western blot (Supplementary Figure S4). (**E** and **F**) IRF3-deficient HEK293T cells were reconstituted with IRF3 WT or R285Q and stimulated with MAVS or STING. Cell lysates were subjected to denaturing (E) or native PAGE (F) followed by immunoblotting with anti-V5 for total IRF3 (E and F), anti-IRF3 pS386, anti-STING or anti-Vinculin as a control (E).

**Figure 4.**
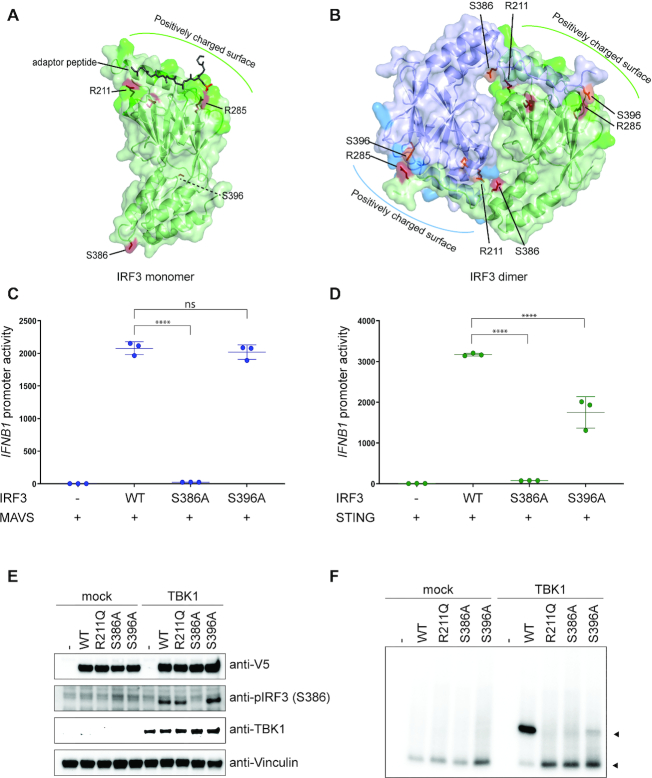
The role of residue R211 in IRF3 dimerization. (**A** and **B**) Structural representation of IRF3 monomer (A) or dimer (B), where IRF3 is shown in green and blue, bound adaptor peptide is shown in dark green (A) and residues are highlighted in red and orange. (A and B) Figure was modeled on PDB ID: 5JEJ (A) and 5JEM (B). (**C** and **D**) IRF3-deficient HEK293T cells were transiently transfected with *IFNB1* promoter firefly luciferase reporter, a constitutively active *Renilla* luciferase reporter and either WT IRF3 or mutant IRF3 as indicated. The cells were stimulated with MAVS (C) or cGAS and STING (D) and luciferase activities were measured 24 h post transfection. Firefly luciferase activity was normalized to Renilla luciferase activity and presented as triplicates ±SD. One-way ANOVA test was used for statistical analysis. ns, not significant; ****, *P* ≤ 0.0001. Similar data were obtained for two independent experiments (Supplementary Figure S6). (**E** and **F**) IRF3-deficient HEK293T cells were reconstituted with IRF3 WT or mutated IRF3 as indicated and stimulated with TBK1. Cell lysates were subjected to denaturing (E) or native PAGE (F) followed by immunoblotting with anti-V5 for total IRF3 (**E** and **F**), anti-IRF3 pS386, anti-STING or anti-Vinculin as a control (E).

**Figure 5.**
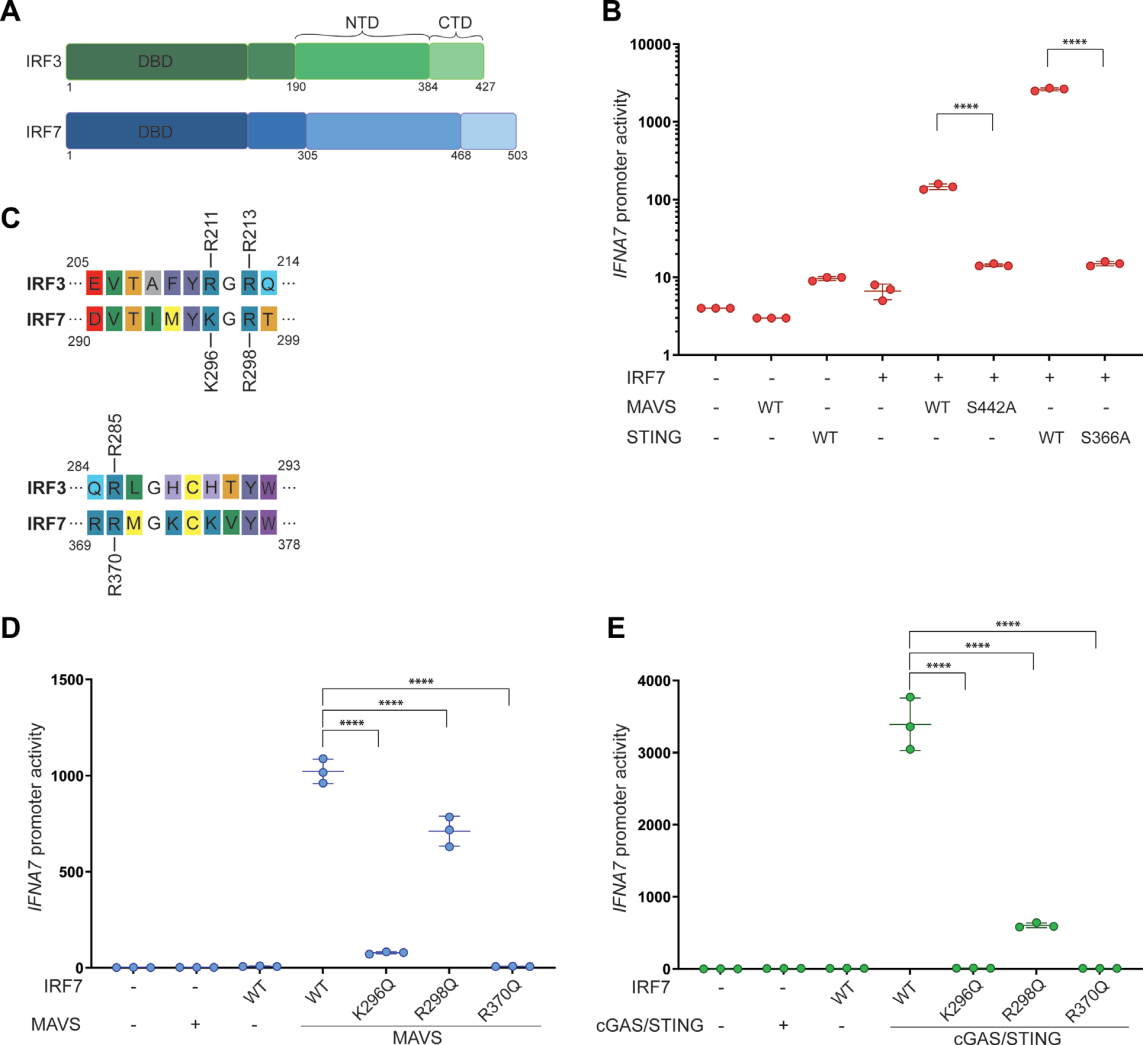
IRF7 docks to MAVS and STING in a similar manner as IRF3. (**A**) Schematic representation of the domain structure of IRF3 (green) and IRF7 (blue). NTD: N-terminal domain, CTD: C-terminal domain. (**B**) IRF3-deficient HEK293T cells were transiently transfected with *IFNA7* promoter firefly luciferase reporter, a constitutively active *Renilla* luciferase reporter and WT IRF7. The cells were stimulated with either MAVS WT or MAVS S442A or cGAS and either STING WT or STING S366A and luciferase activities were measured 24 h post transfection. Firefly luciferase activity was normalized to *Renilla* luciferase activity and presented as triplicates ±SD. One-way ANOVA test was used for statistical analysis. ns, not significant; *, *P* ≤ 0.05; **, *P* ≤ 0.01; ***, *P* ≤ 0.001. Similar data were obtained for two independent experiments. (**C**) Sequence alignment of IRF3 and IRF7. A representative section of the alignment is depicted to show conservation of residues. (**D** and **E**) IRF3-deficient HEK293T cells were transiently transfected with *IFNA7* promoter firefly luciferase reporter, a constitutively active *Renilla* luciferase reporter and either WT IRF7 or mutated IRF7 as indicated. The cells were stimulated with either MAVS or cGAS and STING and luciferase activities were measured 24 h post transfection. Firefly luciferase activity was normalized to *Renilla* luciferase activity and presented as triplicates ± SD. One-way ANOVA test was used for statistical analysis. ****, *P* ≤ 0.001. Similar data were obtained for two independent experiments. Protein expression was analyzed by western blotting (Supplementary Figure S6).

**Figure 6.**
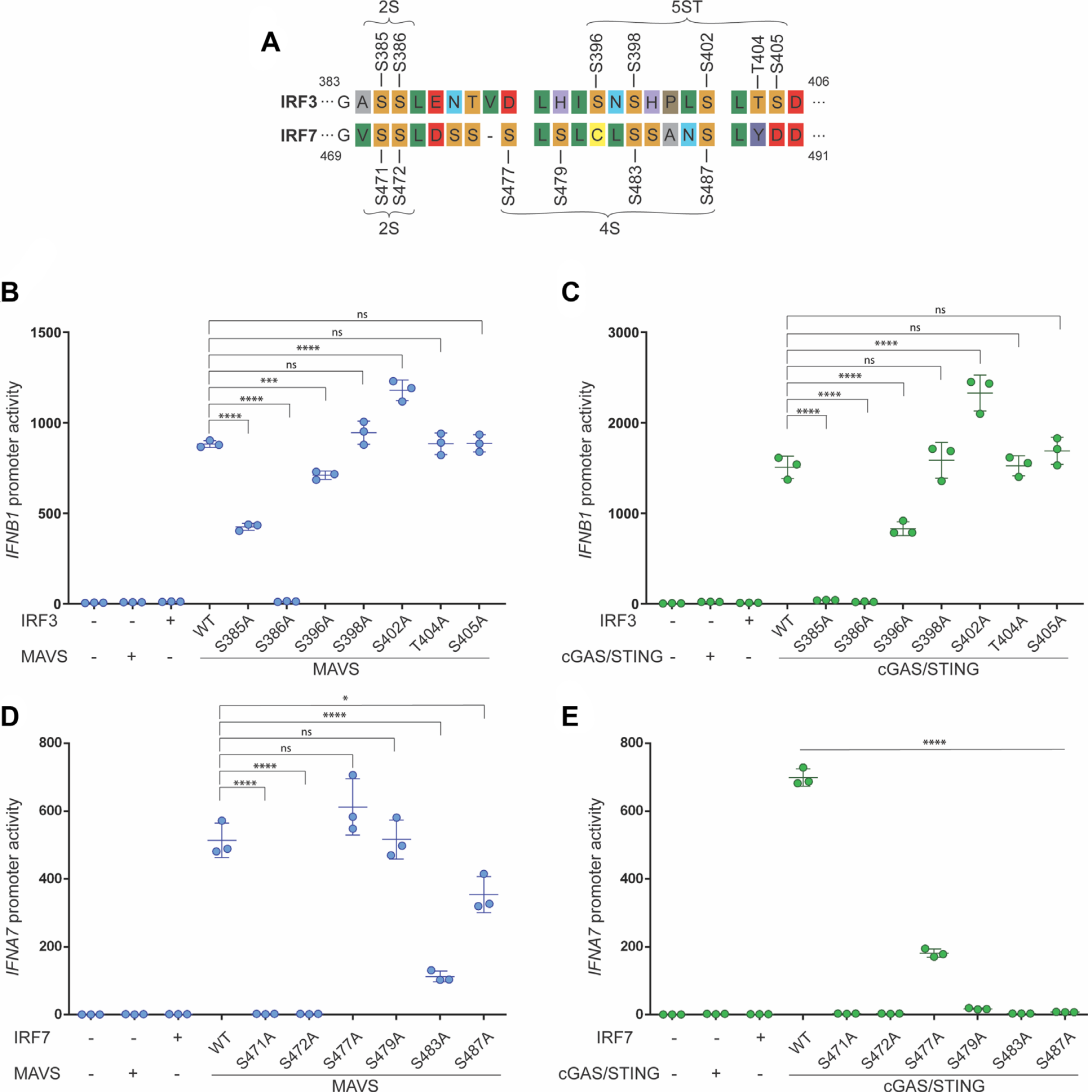
IRF7 requires additional phosphorylation events for activation. (**A**) Sequence alignment of IRF3 and IRF7. A representative section of the alignment is depicted to show conservation of residues. (**B–E**) IRF3-deficient HEK293T cells were transiently transfected with *IFNB1* promoter firefly luciferase reporter (B and C) or *IFNA7* promoter firefly luciferase reporter (D and E) and a constitutively active *Renilla* luciferase reporter. (B and C) The cells were transfected with either WT IRF3 or mutated IRF3 as indicated and stimulated with either MAVS or cGAS and STING. (D and E) The cells were transfected with either WT IRF7 or mutated IRF7 as indicated and stimulated with MAVS or cGAS and STING. (B–E) Luciferase activities were measured 24 h post transfection. Firefly luciferase activity was normalized to *Renilla* luciferase activity and presented as triplicates ± SD. One-way ANOVA test was used for statistical analysis. ns, not significant; *, *P* ≤ 0.05; ****, *P* ≤ 0.0001. Bar in (E) shows significance for all mutations tested. Similar data were obtained for two independent experiments. Protein expression was analyzed by western blotting (Supplementary Figure S7).

HEK293T cells were seeded at a density of 4 × 10^5^ cells/ml in 12-well plates and cultured for 24 h. The cells were transiently transfected using a mixture of DNA and polyethylimine (PEI) (Polyscience) in a ratio of 1:3. The DNA mixture consisted of 970 ng firefly luciferase reporter plasmid, 30 ng reporter plasmid containing a *Renilla* luciferase gene under the control of a constitutive active promoter and 1000 ng plasmids of interest. The firefly reporter plasmids used contained either the *IFNB1* promoter (Figures 1, 3 and 4), the first 55 base pairs of the *IFNB1* promoter fused to several repeated IRF3-binding sites (Figure 2), several repeats of NF-kB binding sites (Figure 2) or the *IFNA7* promoter (Figures 5 and 6). For all experiments 50 ng of either WT IRF3 or mutated IRF3 plasmid was used. For stimulation with MAVS either 250 ng (Figures 1, 2, 4, 5 and 6) or 50 ng (Figure 3) plasmid was used. For stimulation with TRIF 50 ng plasmid was used. For stimulation with STING, either 50 ng STING and 10 ng cGAS (Figure 1) or 100 ng STING and 20 ng cGAS (Figures 2–6) was used. Empty pcDNA3.1 plasmid was added to give a total of 2000 ng DNA per well. Cells were lysed after 24 h using Passive Lysis Buffer (Promega) and the luciferase activity was measured with Dual-Luciferase Reporter Assay System (Promega) following the manufacturer’s instructions.

### Western blotting

The cell lysates were run on a 10% SDS polyacrylamide gel and transferred to a PVDF-Star transfer membrane (AppliChem). The amount of IRF3 and IRF7 in the cell lysates was detected using a mouse anti-V5 antibody (1:5000, Invitrogen), MAVS and cGAS was detected with a mouse anti-FLAG antibody (1:2000, Sigma Aldrich), STING was detected with a rabbit anti-STING antibody (1:1000, Cell signaling), pTBK-1 was detected with a rabbit anti‐pTBK1 (Ser172) (1:1000, Cell Signaling), vinculin was detected with anti-vinculin (Sigma) and the amount of GAPDH was detected using a rabbit anti-GAPDH antibody (1:1000, Santa Cruz Biotechnology). The primary antibodies were followed by either HRP-conjugated anti-mouse antibody (Jackson Immuno research) for the anti-V5 antibody or HRP-conjugated anti-rabbit (Dako Cytomation) for the anti-GAPDH antibody. The western blot was developed using SuperSignal West Dura extended Duration substrate (Thermo Scientific) and visualized on an X-ray film (Konica Minolta).

### Native PAGE

HEK293T cells were transfected with empty vector, IRF3 WT or IRF3 mutants. The cells were stimulated by co-transfection with TBK1 or with MAVS or by infection with SeV. Cells were lysed in lysis buffer (1% NP-40, 50 mM Tris-Cl pH 8.0, 150 mM NaCl, 1 mM vanadate and 1 mM PMSF) and subjected to native PAGE followed by immunoblotting with antibodies against V5 and human IRF3 S386-P (18783) from Immuno-Biological Laboratories Co. Ltd. (14).

### Statistical analysis

The data are shown as mean ± SD, either from biological triplicates as indicated and the experiments were repeated as indicated. For comparison of multiple groups, statistical significance was determined using one‐way ANOVA and Tukey multiple comparisons method was used when comparing the mean of one group to the mean of any other group. All statistical analyses were performed using the GraphPad Prism 7.02 software.

## RESULTS

### Identification of IRF3 surface residues important for activation

Previous work by Chen and associates identified positively charged (also referred to as basic) patches on IRF3 playing a key role in docking to the adaptors and subsequent dimerization (17). In the present work, we are exploring the role that these amino acids play individually and if there are adaptor-specific interactions with IRF3. To test this, we mutated each of the basic surface residues R211, R213, R255, R262, H263, R285, H288, H290, K313, K315, K360 and R361 on IRF3 to glutamine. We chose mutation to glutamine since glutamine carries a similar size and hydrophobicity but is not charged and therefore unable to form specific interactions with phosphates. To evaluate the effect of these mutations on *IFNB1* promoter activation, we utilized IRF3-deficient HEK293T cells and co-expressed each of the three adaptor molecules MAVS, TRIF and STING. Transfection of MAVS or TRIF alone drives strong *IFNB1* promoter activation, whereas activation of STING-dependent IFN expression in HEK293T cells requires co-transfection with low amounts of cGAS (22).

The screening identified two residues of interest: R211 and R285. The mutation of R211 to Q resulted in abolished *IFNB1* promoter induction for all three adaptor stimulations, whereas the mutation of R285 to Q resulted in impaired activation through TRIF and STING but to a lesser extent through MAVS, in agreement with previous observations (19) (Figure 1A–C).

To validate if the phenotype observed for these mutants was unique to HEK293T cells, we utilized IRF3-deficient THP-1 monocytes. In THP-1 cells, SeV is sensed through the RIG-I–MAVS pathway (23,24) and HSV-1 through the cGAS–STING pathway (25). HSV-1 can also activate the TLR3–TRIF pathway; however, TLR3 is only expressed in very low levels in THP-1 cells and therefore appears to be of less importance (26). Thus, in the following we are focusing on the cytosolic pathways of cGAS–STING and RIG-I–MAVS. The THP-1 cells were reconstituted with either wild type (WT) or mutated IRF3 by lentiviral transduction (Supplementary Figure S2) and then differentiated to macrophages by phorbol 12-myristate 13-acetate (PMA) treatment. The cells received three different treatments; SeV, dsDNA and HSV-1. After stimulation, the RNA was harvested and analyzed for IFNβ mRNA expression by qPCR. In agreement with the data obtained in the HEK293T cell-based system, the IRF3 R211Q mutant resulted in complete loss of *IFNB1* induction independently of the type of stimulation (Figure 1D). The IRF3 R285Q exhibited some residual activity with SeV infection but not when stimulated through the cGAS–STING pathway (Figure 1D), again in agreement with the data obtained in HEK293T cells (Figure 1A, C). Overall, these data suggest a crucial role for residue R211 in IRF3 activation, whereas the role of R285 is partly redundant if activation occurs through MAVS, but required if activation occurs through STING.

### Does strong NF-κB activation by MAVS partially compensate for the defect in IRF3 R285Q?

So far, the effect of mutating R285 in IRF3 was evaluated solely by the induction of the *IFNB1* promoter, either using a reporter construct or by measuring endogenous IFNβ mRNA. However, the *IFNB1* promoter contains both IRF3- and NF-κB-binding sites. To investigate if MAVS indirectly compensates for the defect in the IRF3 R285Q mutant by a stronger NF-κB response, we performed two experiments. First, we used an artificial IRF3 reporter, where the first 55 base pairs of the *IFNB1* promoter were fused to several repeated IRF3 binding sites. This reporter construct does not contain any NF-κB-binding sites and depends only upon IRF3 for transcription. In this experiment, the R285Q mutation exhibited similar effects on MAVS and STING activated IRF3 as achieved using the full *IFNB1* promoter (Figure 2A, B). As a control, we used a reporter construct where the luciferase expression was driven by eight repeats of NF-κB-binding sites. Similar NF-κB activities were achieved for MAVS and STING stimulation and importantly no decrease in NF-κB induced reporter activation was observed for the R285Q version of IRF3 (Figure 2C, D). Thus, the differences observed in the activity of IRF3 R285Q after activation with MAVS as opposed to STING is not due to an indirect effect caused by differential NF-κB activation by the two adaptors.

### The role of residue R285 in IRF3 docking to the adaptors MAVS and STING

To explain the observed effect of R285Q, we examined the previously published structures of IRF3 bound to peptides originating from either MAVS, TRIF or STING containing the pLxIS motif (21). Residue R285 is situated opposite the phosphoserine in the pLxIS motif (Figure 3A). The flat guanidinium group of arginine forms a strong electrostatic interaction with the tetrahedral phosphate, suggesting that R285 is critical for interaction of IRF3 with the phosphorylated adaptor proteins. However, this does not explain why activation through the MAVS pathway is less sensitive to the R285Q mutation in IRF3 than activation through the STING pathway. Therefore, we decided to test if phosphorylation of the pLxIS motif in MAVS is critical for activation of IRF3. We mutated S442 in the pLxIS motif of MAVS to alanine and transiently transfected MAVS-deficient HEK293T cells with increasing amounts of either MAVS WT or MAVS S442A (Figure 3B). The mutation exhibited a slight increase in IRF3 activity, and this observation was independent of the amount of MAVS transfected into the cells (Figure 3B). However, the S442A mutation of MAVS appeared to express moderately higher level of protein than the WT version of MAVS, which could possibly explain the slight increase in activity (Supplementary Figure S4A). Next, we examined the effect of S442A (MAVS) in the context of R285Q (IRF3) in IRF3-deficient HEK293T cells. The combination of R285Q (IRF3) and S442A (MAVS) led to a significant decrease in IRF3 activity compared to the combination of S442A (MAVS) with WT IRF3 (Figure 3C). This synergistic effect of the two mutations is in agreement with the fact that R285 forms a specific interaction with the phosphoserine on the adaptor proteins. Thus, while R285 in IRF3 clearly interacts with the phosphorylated S442 in MAVS, this interaction is not required for IRF3 activation. Finally, when mutating the corresponding serine in the STING pLxIS motif, the activation of WT IRF3 was severely impaired and even more so when combining the STING pLxIS mutant with either of the IRF3 mutants (Figure 3D).

To directly evaluate the effect of the R285Q mutation upon IRF3 phosphorylation, we performed western blot analysis using an antibody directed against the phosphorylated form of S386 in IRF3 (Figure 3E). This revealed that both MAVS and STING expression lead to phosphorylation of WT IRF3 but not the R285Q version, confirming our previous finding (19). However, western blot is not sufficiently sensitive to measure residual/minimal IRF3 R285Q phosphorylation by MAVS. Finally, we also performed IRF3 dimerization assays (Figure 3F), and found little dimerization of the IRF3 R285Q mutant. As phosphorylation is a prerequisite for dimerization this was expected.

Collectively, these data suggest that the phosphorylated serine in the STING pLxIS motif plays a crucial role in the interaction with IRF3. However, while the specific interaction of IRF3 with the equivalent serine in the MAVS pLxIS motif does occur, it is not essential for activation of IRF3 in HEK293T cells. Furthermore, the mutation of R285 to Q in IRF3 led to decreased phosphorylation and dimer formation of IRF3.

### R211 is critical for interaction with phosphorylated S386 in the active dimer of IRF3

Our data show that R211 is critical regardless of the adaptor used for activation. Furthermore, structural data do not show any strong interaction between R211 and the adaptor proteins (Figure 4A), except for TRIF where there is a water mediated interaction between the phosphorylated S202 (TRIF) and R211 (IRF3). An equivalent of S202 is not found in the other adaptor proteins and while this relatively weak interaction may play a role in TRIF-mediated interaction of IRF3, it cannot be responsible for the phenotype seen with MAVS and STING. In contrast, the structure of the IRF3 dimer shows a clear salt bridge between R211 and the phosphorylated S386 (Figure 4B). This led us to hypothesize that R211 is not involved in a critical interaction with the pLxIS motif but is instead required for IRF3 dimerization by forming a salt bridge with phosphorylated S386 (Figure 4A,B). We started by evaluating which phosphoserine is required for activation of IRF3 by mutating both S386 and S396. This showed that S386 is required for IRF3 activation whereas mutating S396 to alanine had no effect when stimulating with MAVS and a minimal effect when stimulating with STING in HEK293T cells (Figure 4C, D; Supplementary Figure S5).

We also tested phosphorylation of IRF3 by western blotting using an antibody specific for phosphor-serine at position 386. This showed that the R211Q mutant, the S396A mutant and WT IRF3 were phosphorylated at S386 (Figure 4E). Finally, in order to directly test dimerization of WT IRF3 and the mutants, we activated IRF3 by overexpression of TBK1 and performed a native PAGE to detect IRF3 dimer formation. WT IRF3 formed a strong dimer whereas no dimerization was detected for either R211Q or S386A (Figure 4F). We could detect a clear IRF3 dimer for S396A; however this was weaker than what was seen for WT IRF3 (Figure 4F). In conclusion, these data show that R211Q is phosphorylated but unable to form a stable dimer thus supporting the hypothesis presented above.

### Mechanism of IRF7 activation and dimerization

Sequence alignment suggests that IRF7 has a similar domain architecture to IRF3 and thus suggests that IRF7 is activated in a similar manner (Figure 5A). However, currently there are neither structural information available for the regulatory domain of IRF7, nor direct evidence for IRF7 activation through the STING and MAVS pathways. First, we wanted to clarify whether STING and MAVS lead to direct activation of IRF7. To do this, we used the HEK293T IRF3 KO cells since these cells do not express IRF7 in any detectable amount (27). In these cells, both MAVS and STING could signal in an IRF7 dependent manner (Figure 5B). In contrast to our observation for IRF3, IRF7 required the serine S442 for MAVS signaling as well as the S366 for STING signaling (Figure 5B). Thus, both MAVS and STING can activate IRF7 in a manner requiring the serine in the pLxIS motif. In IRF7, R211 is substituted by a lysine (K296), but R285 is fully conserved (R370) (Figure 5C). Since R211 was not completely conserved the neighboring arginine was also tested. Our experiment shows, that the equivalent for R211 and R285 are required for IRF7 activity (Figure 5D, E). In addition, R298Q (equivalent of R213 in IRF3) led to approximately 50% reduction of IRF7 activity where the equivalent residue in IRF3 had little or no effect (Figure 5D, E).

### IRF7 requires additional phosphorylation events for activation

IRF3 and IRF7 have similar phosphorylation sites within the C-terminal domain, where IRF3 has a 2S and a 5ST site and IRF7 has a 2S and a 4S site (12,28). Phosphorylation patterns of IRF3 and IRF7 were previously investigated, but only by multiple mutations of the whole phosphorylation site. For this study, we wanted to investigate the individual contribution of each residue within the 2S and the 5ST/4S sites to the activity of IRF3 and IRF7, respectively. Figure 6A shows a summary of a sequence alignment highlighting potential phosphorylation sites of IRF3 and IRF7. We mutated the putative phosphoserines in both IRF3 and IRF7 and investigated the effect of these mutations upon activation with either MAVS or STING in HEK293T IRF3 KO cells. This highlighted an interesting difference between IRF3 and IRF7, where IRF3 only needs phosphorylation in the 2S site (S386) for activity, whereas IRF7 needs at least one phosphorylation event in the 4S site, in addition to phosphorylation of both the residues at the S2 site.

Our data show that the only essential phosphorylation event for IRF3 was S386. Mutation of individual serines in the second site had no significant effect on IRF3 activity when using MAVS as the activator, whereas a marginal loss of activity was seen for the S396A mutation using STING as the activator. Furthermore, phosphorylation of S385 was critical when using STING as the activator but mutation of this residue only lead to 50% loss of activity when stimulating using MAVS (Figure 6B, C). In contrast, mutation of S471, S472 or S483 in IRF7 led to a total loss of IRF7 activity for both types of stimulation (Figure 6D, E). Thus, in contrast to IRF3, IRF7 requires phosphorylation in both clusters to become transcriptionally active suggesting that IRF7 activation is more stringently controlled than that of IRF3.

## DISCUSSION

The current model for IRF3 activation dictates that the activated innate adaptor proteins MAVS and STING are phosphorylated at their pLxIS motif at positions S442 and S366, respectively. This creates a docking site for IRF3, where a positively charged surface on IRF3 interacts with the phosphorylated innate immune adaptors. Our data demonstrate that both IRF3 and IRF7 can interact with MAVS and STING and that this docking involves critical interactions between the serine at the pLxIS motif of the adaptor and residue R285 or R370 in IRF3 and IRF7, respectively. The docking enables phosphorylation and thereby activation of both IRF3 and IRF7 in a MAVS and STING dependent manner. However, our data also underline an important exception from this model, where MAVS mediated activation of IRF3 can occur in the absence of a phosphoserine in the pLxIS motif. This observation agrees with previous work from us, where we identified an IRF3 R285Q mutation in a patient who was predisposed towards infection with DNA virus but not towards infection with RNA virus (19). Based on our findings, we present a revised and extended model for IRF3 and IRF7 docking to innate immune adaptors, the subsequent phosphorylation of IRF3 and IRF7 and the formation of the transcriptionally active homodimers.

Our revised model for activation of IRF3 and IRF7 helps us understand why both MAVS and STING create ‘signaling hubs’ generated by multimerization of these proteins (29,30). Upon phosphorylation, IRF3 has to leave the adaptor as a monomer and the recently added phosphorylation will be exposed to cellular phosphatases. However, when phosphorylation occurs within these ‘signaling hubs’, IRF3 is likely to meet a similar phosphorylated IRF3 molecule, which allows for the formation of the stable homodimer where the phosphoserine is no longer accessible to phosphatases. Thus, the use of ‘signaling hubs’ has an inherent protection against accidental activation by random phosphorylation, since a randomly phosphorylated IRF3 molecule will be less likely to meet a suitable partner and thus be less likely to initiate an inappropriate IFN response.

The effect we observed when mutating S442A in MAVS has been addressed before in Liu *et al.* (17). These authors found that introducing the MAVS S442A mutant in MAVS deficient mouse embryonic fibroblasts infected with vesicular stomatitis virus (VSV) led to an abrogated IFNβ production. This observation is not in complete agreement with our data, but it is worth noting that the data from Liu *et al.* were obtained in a different species and cell type and with a different expression system then the data generated here. Furthermore, we observed an effect of mutating S442 in MAVS when investigating IRF7 activation, something which was not investigated by Liu *et al.* How MAVS achieves activation of IRF3 in the absence of phosphorylation at S442 is currently not clear to us, but we believe it is likely to involve the multimerization of MAVS. MAVS forms prion-like filaments in large aggregates that would create large activation platforms for interaction with IRF3 and other signaling proteins (31).

Upon docking to the phosphorylated adaptor proteins, IRF3 is poised for phosphorylation by TBK1 which is associated with the adaptor proteins. The positively charged region of IRF3, responsible for docking to the adaptor, is found at the opposite end of the IRF3 molecule than the phosphorylation sites, which renders these sites accessible for kinases, like TBK1. The positively charged surface of IRF3 is thus involved in two distinct interactions that are the docking of IRF3 to the adaptors and the subsequent formation of the IRF3 homodimer. These interactions are governed by distinct residues of IRF3. The R285 of IRF3 is forming the critical interaction with the phosphoserine of the adaptor proteins while the interaction between R211 and the phosphorylated S386 is critical for the formation of the transcriptionally active IRF3 homodimer. Interestingly, we did observe a tendency toward higher expression of the ‘dead’ mutants of both IRF3 and IRF7, as compared to wild-type. Further studies will reveal if this is caused by an activation mediated degradation of IRF3 and IRF7.

Although activation of IRF3 and IRF7 occurs by similar mechanisms, there are important differences. First, activation of IRF7 fully depends upon the serine in the pLxIS motive of MAVS, whereas IRF3 can achieve at least partial activation even if this residue is mutated to alanine. In agreement with this, R370 (equivalent to R285 in IRF3), which forms the interaction with the phosphoserine in the pLxIS motif of the adaptors, was found to be absolutely required for IRF7 activity. Second, IRF3 only requires phosphorylation of the 2S site for activation, whereas IRF7 requires phosphorylation of both the 2S and 4S sites for activation.

Those interesting observations raise the question of the molecular mechanism underlying the more strict regulation of IRF7. The first question is if all phosphorylations are performed by the same kinase and while we think it is highly likely that S471 and S472 are phosphorylated by the same kinase, it is an open question if phosphorylation in the 4S site occurs via the same or a different kinase. Unfortunately, we had technical issues with detection of phosphorylated forms of IRF7 by both western blot analysis and using advanced mass spectrometry approaches. Thus, we need better technologies to detect specific IRF7 phosphorylations before answering this question.

The second question, which is raised by our observations, is why IRF7 needs phosphorylation at the 4S site when IRF3 does not? We have considered at least two quite different explanations for this. Based upon the structure of the IRF3 dimer, it is quite possible that the dimer is stabilized by phosphorylation in the 5ST site of IRF3, and when we mutate S396 of IRF3, we do see less dimer formation (Figure 4F), thus IRF7 might depend upon phosphorylation of the 4S site in order to form a stable dimer. An alternative explanation is that phosphorylation of the 4S site in IRF7 facilitates phosphorylation of the 2S site either by facilitating adaptor docking or by exposing the S2 site to kinases. The fact that mutations in the 4S site of IRF7 have quite different phenotypes for MAVS and STING mediated activation argues in favor of the latter explanation. Finally, it is quite possible that both modes act in combination. Thus, substantial more work is needed in this area, including high-resolution structures of IRF7.

While the molecular basis for the more tight regulation of IRF7 needs further investigation, biologically it appears rational that activation of IRF7 is more stringently controlled. IRF7 activation can lead to production of large amounts of IFN-α, which again leads to a strong and systemic IFN response, which is associated with significant immune pathology (32) as opposed to IRF3 driven IFNβ expression.

## Supplementary Material

gkaa873_Supplemental_File

## Notes

Present address: Line L. Andersen, Institute of Virology, Technical University of Munich, Munich, Germany.
