# Supplementary material for: Characterization of distinct molecular interactions responsible for IRF3 and IRF7 phosphorylation and subsequent dimerization
Source: Nucleic Acids Res. 2020 Oct 22;48(20):11421–33. doi: 10.1093/nar/gkaa873 (PMC7672473; doi:10.1093/nar/gkaa873)

Supplementary figures

Figure S1: Replicated experiments for Figure 1. (A-C) IRF3-deficient HEK293T cells were transiently transfected with *IFNB1* promoter firefly luciferase reporter, a constitutively active *Renilla* luciferase reporter and either WT IRF3 or mutant IRF3 as indicated. The cells were stimulated with MAVS (A), TRIF (B) or STING and cGAS (C) and luciferase activities were measured 24 h post transfection. Firefly luciferase activity was normalized to *Renilla* luciferase activity and presented as percentage activity of WT IRF3 with grand mean.

Figure S2: Transduced IRF3 levels in IRF3-deficient THP-1 cells. IRF3-deficient THP-1 cells transduced with V5-tagged IRF3 WT, IRF3 R211Q, IRF3 R285Q or IRF3 R361Q were infected for 6 h with SeV (2 HAU/mL) or HSV-1 (MOI 9) or stimulated for 6 h with 1 μg dsDNA. Total RNA was harvested and subjected to RT-qPCR for measurement of V5-tagged IRF3 specifically. (A) IRF3 mRNA levels are shown as triplicates ±SD. Similar data was obtained for 2 independent experiments. (B) Protein level was analyzed by western blotting with anti-V5 antibody for detection of IRF3. GAPDH was detected as a loading control.

Figure S3: Protein expression for Figure 2. (A-D) Protein levels for Figure 2 (A-D) were analyzed by western blotting with anti-V5 antibody for detection of IRF3, anti-FLAG for detection of MAVS and anti-STING for detection of STING. GAPDH was detected as a loading control.

Figure S4: Replicated experiments and protein expression for Figure 3. (A) MAVS-deficient HEK293T cells were transiently transfected with varying amounts of either MAVS WT or MAVS S442A. Protein levels were analyzed by western blotting with anti-FLAG for detection of MAVS protein and anti-GAPDH as loading contrl. (B, C) IRF3-deficient HEK293T cells were transiently transfected with *IFNB1* promoter firefly luciferase reporter, a constitutively active *Renilla* luciferase reporter and either WT IRF3 or mutant IRF3 as indicated. The cells were stimulated with either MAVS WT or MAVS S442A (B) or cGAS and either STING WT or STING S366A (C) and luciferase activities were measured 24 h post transfection. Firefly luciferase activity was normalized to *Renilla* luciferase activity and presented as percentage activity of WT IRF3 with grand mean. Protein levels were analyzed by western blotting with anti-FLAG antibody for detection of MAVS, anti-STING for detection of STING and anti-V5 for detection of IRF3. GAPDH was detected as a loading control.

Figure S5: Replicated experiments and protein expression for Figure 4. IRF3-deficient HEK293T cells were transiently transfected with *IFNB1* promoter firefly luciferase reporter, a constitutively active *Renilla* luciferase reporter and either WT IRF3 or mutant IRF3 as indicated. The cells were stimulated with either MAVS (A) or cGAS and STING (B) and luciferase activities were measured 24 h post transfection. Firefly luciferase activity was normalized to *Renilla* luciferase activity and presented as percentage activity of WT

IRF3 with grand mean. Protein levels were analyzed by western blotting with anti-FLAG antibody for detection of MAVS, anti-STING for detection of STING and anti-V5 for detection of IRF3. GAPDH was detected as a loading control.

Figure S6: Protein expression for Figure 5. (A-C) Protein levels for Figure 5 (B, D, E) were analyzed by western blotting with anti-V5 antibody for detection of IRF3, anti-FLAG for detection of MAVS and cGAS and anti-STING for detection of STING. GAPDH was detected as a loading control.

Figure S7: Protein expression for Figure 6. (A-D) Protein levels for Figure 6 (B-E) were analyzed by western blotting with anti-V5 antibody for detection of IRF3, anti-FLAG for detection of MAVS and cGAS and anti-STING for detection of STING. GAPDH was detected as a loading control.

Figure S1: Replicated experiments for Figure 1

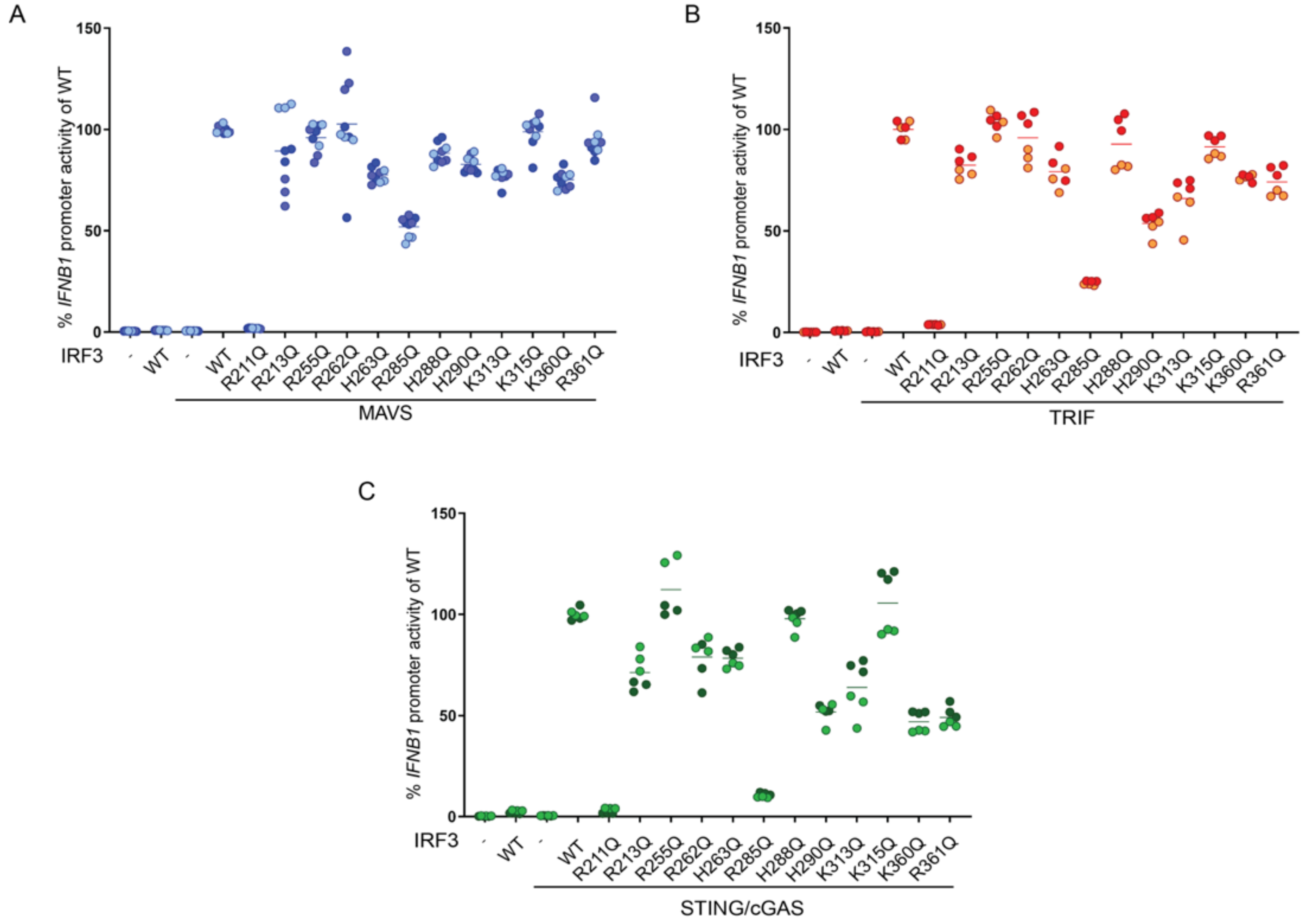

Figure S2: IRF3 expression in transduced IRF3 deficient THP-1 cells in Figure 1

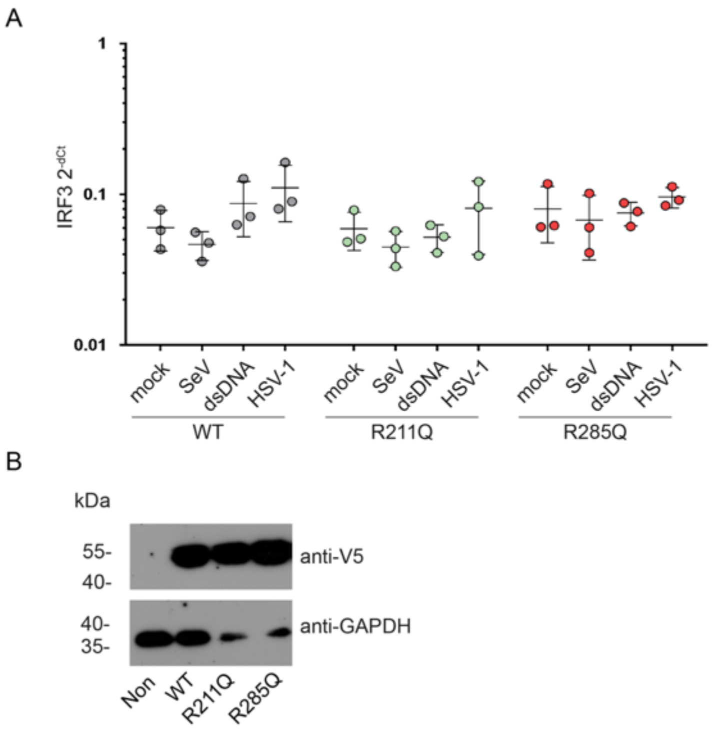

Figure S3: Protein expression for Figure 2

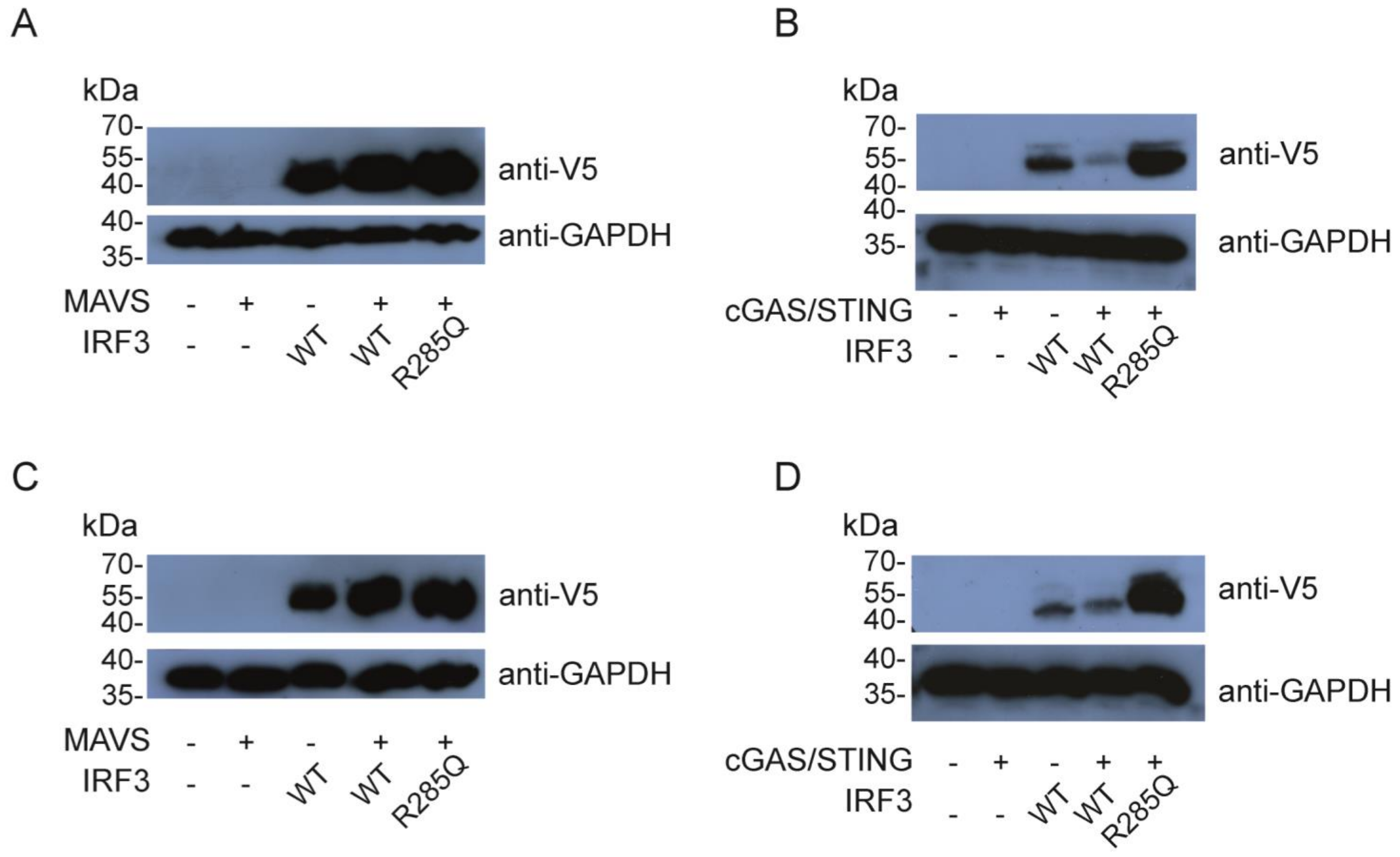

Figure S4: Replicated experiments and protein expression for Figure 3

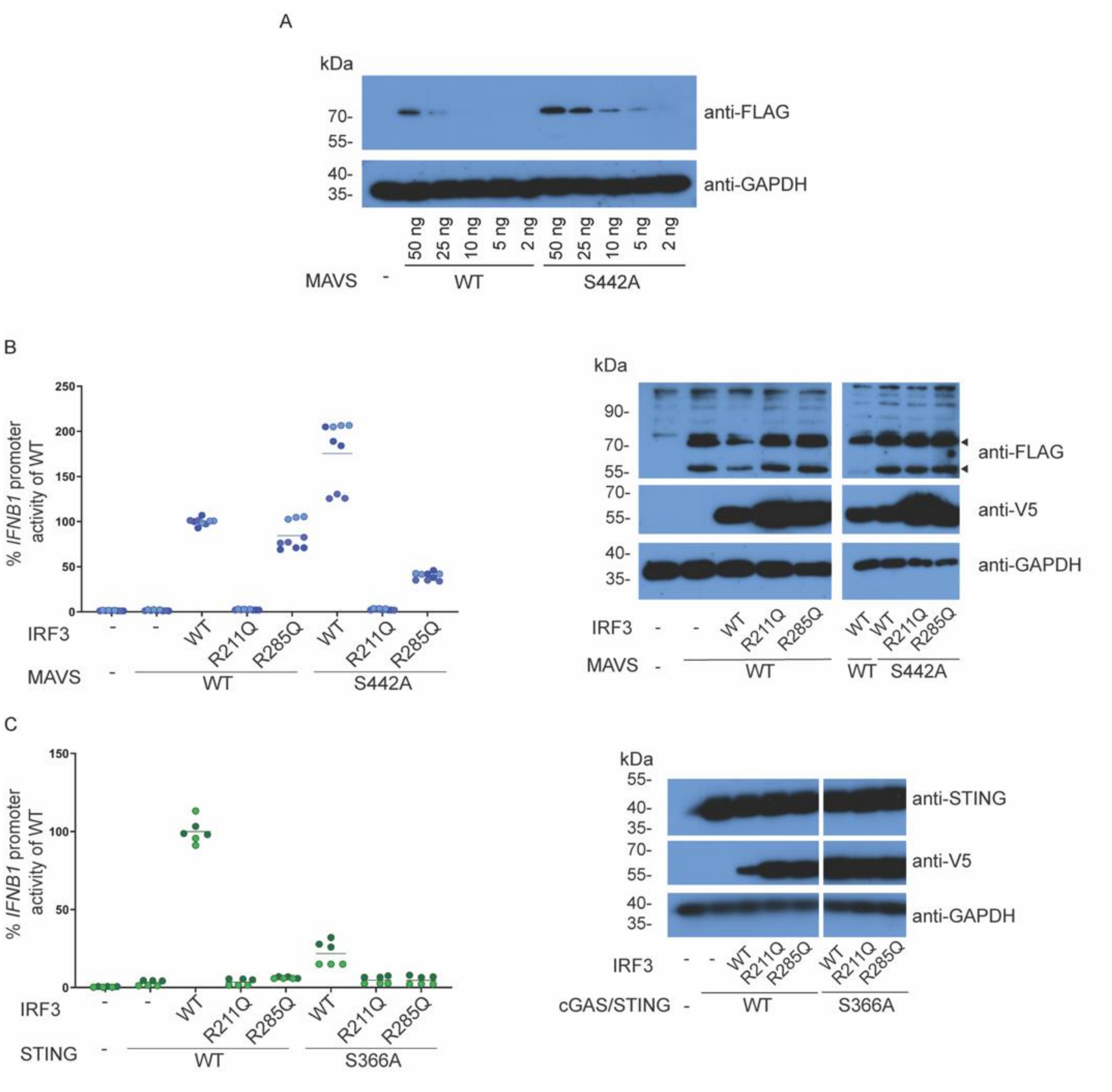

Figure S5: Replicated experiments and protein expression for Figure 4

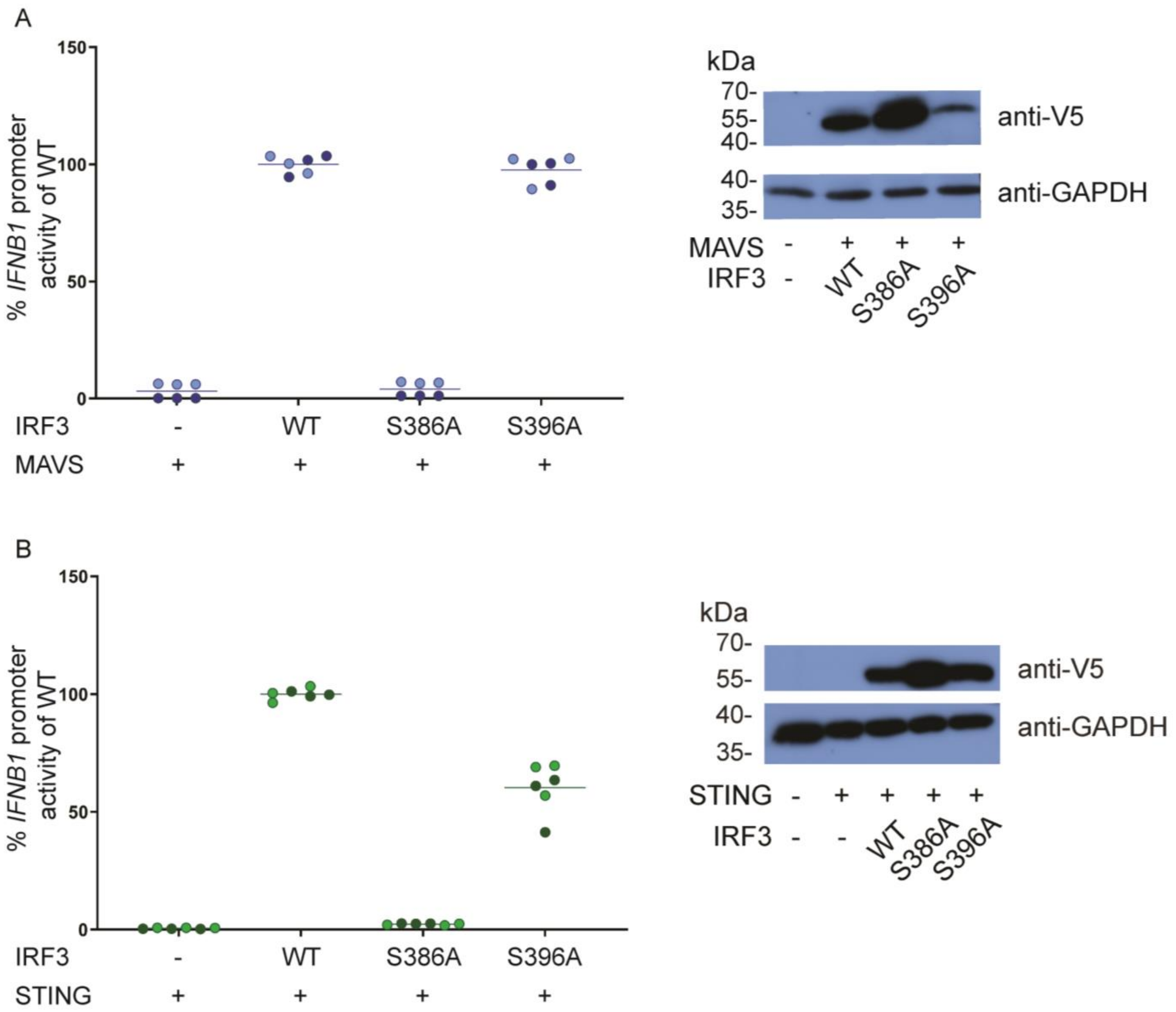

Figure S6: Protein expression for Figure 5

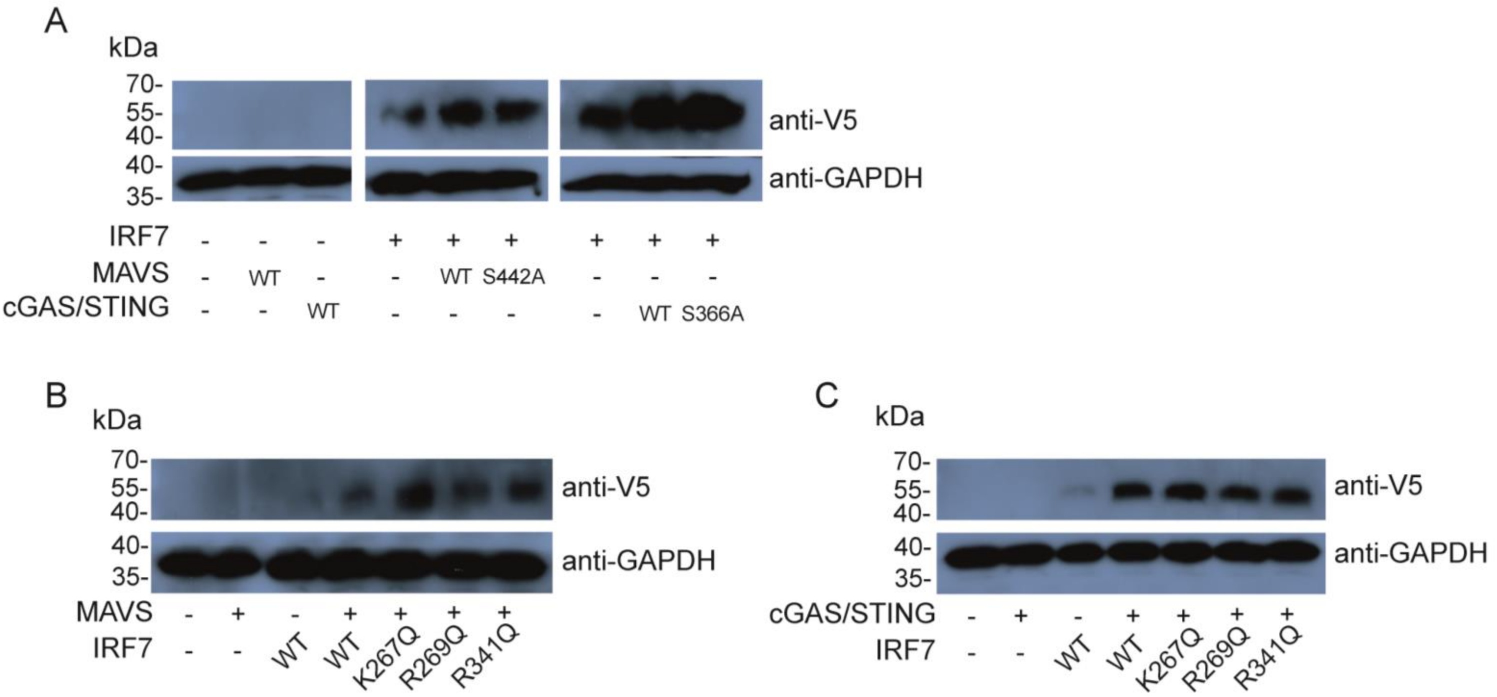

Figure S7: Protein expression for Figure 6

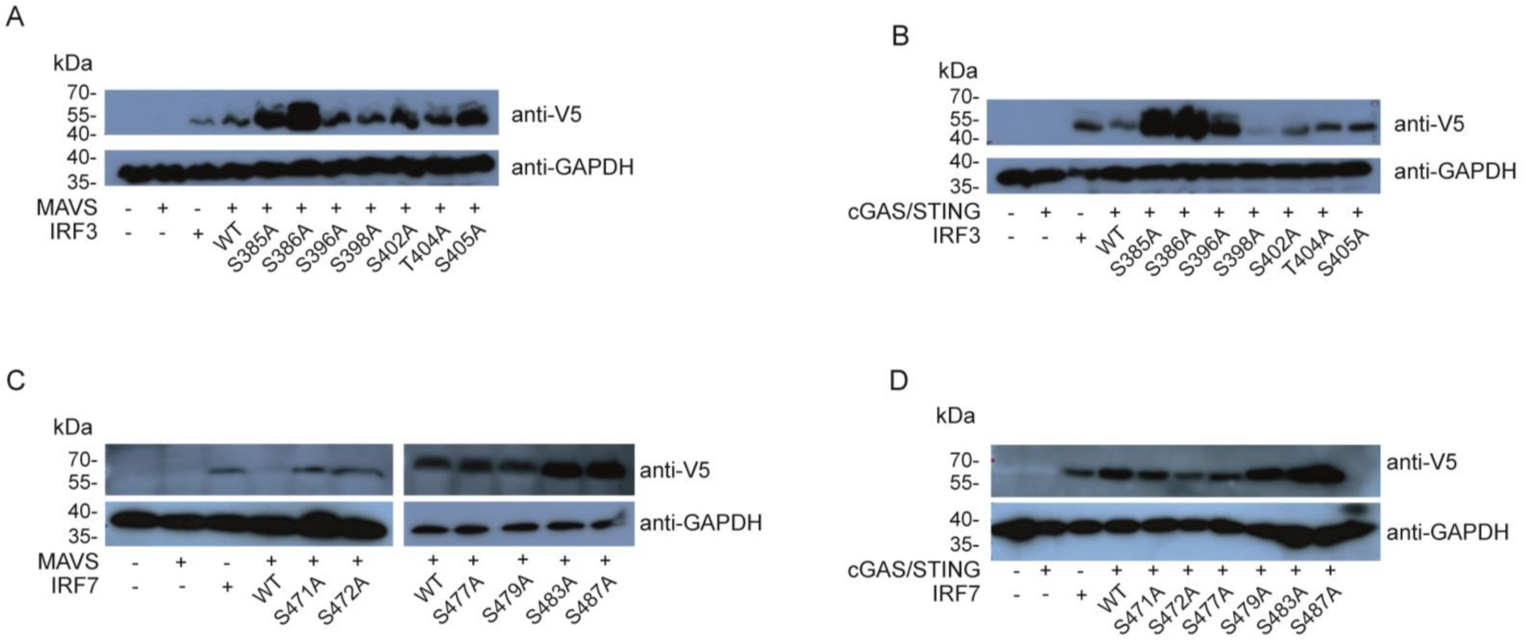

Supplement: gkaa873_Supplemental_File [file gkaa873_supplemental_file.pdf]
